# A low cost neuromorphic learning engine based on a high performance supervised SNN learning algorithm

**DOI:** 10.1038/s41598-023-32120-7

**Published:** 2023-04-18

**Authors:** Ali Siddique, Mang I. Vai, Sio Hang Pun

**Affiliations:** Department of Electrical and Computer Engineering, Faculty of Science and Technology, University of Macau, Taipa, 999078 Macau

**Keywords:** Electrical and electronic engineering, Computational science, Computer science

## Abstract

Spiking neural networks (SNNs) are more energy- and resource-efficient than artificial neural networks (ANNs). However, supervised SNN learning is a challenging task due to non-differentiability of spikes and computation of complex terms. Moreover, the design of SNN learning engines is not an easy task due to limited hardware resources and tight energy constraints. In this article, a novel hardware-efficient SNN back-propagation scheme that offers fast convergence is proposed. The learning scheme does not require any complex operation such as error normalization and weight-threshold balancing, and can achieve an accuracy of around 97.5% on MNIST dataset using only 158,800 synapses. The multiplier-less inference engine trained using the proposed hard sigmoid SNN training (HaSiST) scheme can operate at a frequency of 135 MHz and consumes only 1.03 slice registers per synapse, 2.8 slice look-up tables, and can infer about 0.03$\times {\varvec{10}}^{\varvec{9}}$ features in a second, equivalent to 9.44 giga synaptic operations per second (GSOPS). The article also presents a high-speed, cost-efficient SNN training engine that consumes only 2.63 slice registers per synapse, 37.84 slice look-up tables per synapse, and can operate at a maximum computational frequency of around 50 MHz on a Virtex 6 FPGA.

## Introduction

Artificial neural networks (ANNs) have successfully been used to solve various modern world problems such as facial recognition, health monitoring, and speech recognition^1,2^. However, ANNs are extremely complex and consume a large amount of system energy. This is the motivation behind the development of chips and systems based on spiking neural networks (SNNs), the third-generation neural networks capable of performing complex tasks while consuming a very small amount of energy and area^2–5^. SNNs mimic biological neural networks that are asynchronous in nature and do not perform any operation unless an event occurs. This event-driven behavior makes SNNs suitable for low-energy operation^6,7^. Moreover, since carefully-designed SNNs do not require complex synaptic operations, they can help reduce cost/area of a system. Here, cost is defined as the amount of resources occupied on a field programmable gate arrays (FPGAs) or application-specific integrated circuits (ASICs).

Though SNN inference is way more energy- and cost-efficient than ANN inference, SNN learning is extremely slow and costly. Von-Neumann architectures are, therefore, unsuitable for SNN training^3^. Since graphic processing units (GPUs), general-purpose computers (GPCs) and other such serial processors are unsuitable for SNN acceleration, many researchers have developed high-throughput and energy-efficient SNN processors. One such example is TrueNorth that consumes only 65 mW for processing extremely complex tasks such as facial recognition^3^. Loihi^8^ and SpiNNaker^5^ are two other examples of neuromorphic processors.

Despite the cost- and energy- efficiency of SNNs, SNN training remains a challenging task due to non-differentiability of spikes^9^. Though many SNN training algorithms have been proposed in the literature, most of them are devised only for Von-Neumann systems and are not suitable for dedicated hardware such as FPGAs and ASICs. This is because most of these algorithms involve complex calculations that are quite hard to perform on dedicated architectures. Moreover, these algorithms are generally unsupervised and are based on spike timing, because of which they yield poor accuracy and cannot work for ultra-large networks. Typical examples are spike timing dependent plasticity (STDP), spatio-temporal backpropagation (STBP), SpikeProp and Tempotron^9–12^. Therefore, there is an urgent need to devise highly-accurate, hardware-friendly supervised learning algorithms for SNNs. These algorithms must be suitable for on-chip learning. Not only the algorithms, but the development of efficient hardware SNN engines capable of online learning is the need of the hour.

### Main contributions

This paper presents a novel backpropagation-based training algorithm for SNNs, and a low-cost, high-throughput, hardware-based SNN engine (which uses the proposed algorithm) capable of online learning. The proposed design has been described in Verilog language at the register-transfer level (RTL). The main contributions of this work are as follows:

1. Development of a novel hard-sigmoid-based SNN training (HaSiST) algorithm based on backpropagation. The algorithm is suitable for hardware implementations since it does not require any complex mathematical operations. The algorithm can be implemented on a chip using a few multipliers and adders.
  - HaSiST requires a much smaller time to train SNNs than ReLU-based SNN backpropagation. This is because both hard sigmoid and SNN activation function are sigmoidal in nature and are inter-convertible. The hard sigmoid can easily be converted into a threshold function, by simply maneuvering its steepness. The SNNs trained using rectified linear unit (ReLU), on the other hand, requires a much longer time to accumulate voltage and to match the original ANN in performance. Since the proposed learning scheme ‘HaSiST’ does not require a long training time, the overall energy consumption of the system is significantly reduced.
  - HaSiST does not require weight-threshold balancing, which is a necessary component of typical ReLU-based SNN backpropagation algorithms^13,14^. It does not require error normalization, regularization and/or threshold normalization. Since HaSiST does not require such costly operations, it is easy to use for SNN training and is quite suitable for hardware implementations.
  - Hard Sigmoid can be used even at the output layer without losing accuracy and is quite cheap. ReLU-Softmax schemes such as^14^ use Softmax at the output layer, which is quite costly. ReLU cannot be used at the output layer since ReLU completely cancels out the negative input region and causes the accuracy to degrade. The proposed scheme HaSiST gives good accuracy while being cost-efficient.
2. An asynchronous and event-driven SNN inference engine trained using HaSiST. The engine can process $0.03\times 10^9$ features per second while being extremely cheap.
3. A low-cost online learning engine that uses HaSiST for SNN training. The system processes hidden layers serially in order to reduce cost. However, in order to achieve reasonable throughput, the system processes output neurons in parallel. Moreover, the *train-while-constrain* approach stops overflow/underflow of the intermediate data during the training process, and hence the system cost does not cross a suitable limit.
4. The proposed online learning engine can train both ANNs and SNNs. This is because it uses Hard Sigmoid (HaSi), which is an ANN activation function. The system can perform both ANN and SNN inference, depending on the user’s choice. For SNN inference, all the system has to do is to convert hard sigmoid (HaSi) into a hard threshold.

## Related work and problem definition

Since the proposed scheme HaSiST deals with both algorithm and architecture, we divide our study into these two major parts. We first review various modern SNN training algorithms along-with their merits and demerits. Then, we discuss various neuromorphic hardware designs based on SNNs.

### SNN training algorithms

In recent years, many SNN training algorithms have been proposed in the literature. Most prominent training paradigms from the algorithm side are: spike timing dependent plasticity (STDP), direct training, temporal backpropagation, and ANN-to-SNN conversion.

#### STDP-based schemes

The most traditional method of SNN training is ‘spike timing dependent plasticity (STDP)’ in which the learning takes place on the basis of spike timing between two connected neurons^10,15^. The method is unsupervised in nature, and hence yields low accuracy on typical real-world applications such as digit classification. For such applications, SNN backpropagation yields far higher accuracy than STDP even with a very small network size. Even to achieve a reasonable level of accuracy on moderately large datasets such as MNIST, the STDP requires tens of millions of synapses. Typical example is^16^ in which the STDP-trained SNN achieves only 95% accuracy on MNIST dataset inspite of employing more than 5 millions synapses. Other examples include^17–20^. Not only that, STDP is a complex algorithm that requires a long time to train SNNs and contains a lot of costly operations. STDP requires a lot of exponential terms and multiplications which make it harder to implement.

The training time of an SNN is dominated by the network size, number of time steps, parameter initialization, number of epochs (1 epoch contains multiple time steps), and the training algorithm. As mentioned earlier, the network size in STDP has to be kept extremely large in order to achieve a reasonable accuracy, as in^16^. On the other hand, the work in^21^ requires only 268,800 synapses and easily achieves about 98.7% accuracy since it is based on SNN backpropagation. The STDP is also used in reinforcement learning (RL), where it suffers from similar problems. As demonstrated in^22^ and^23^, even for extremely simple networks, the learning accuracy is in the range of 80% to 90%. The accuracy will definitely degrade for larger networks.

Moreover, the number of time steps required to train an STDP network are generally around 100. For example^24^, requires 700 time steps and achieves only 92.63% accuracy on MNIST with more than 700,000 synapses. Similarly^25^, requires 100 time steps to train an SNN having 130 synapses. The work in^16^ is a similar example. SNN backpropagation, on the other hand, requires fewer time steps and yields higher accuracy. The work in^21^ is a typical example; the system can reach 99.4% accuracy in just 8 time steps.

Recently, though some researchers have developed hardware-efficient STDP systems, none of them is able to compete with hardware SNNs trained using backpropagation (BP). Some of the examples are given in^19,20,25,26^. In^26^, the authors present a hardware SNN processor that achieves only 93% accuracy on MNIST inspite of using 301,056 synapses. The algorithm relies on input truncation and linear STDP that can be implemented easily on hardware. The works in^19,20^ achieve less than 94% accuracy on MNIST inspite of employing 647,000 synapses.

Some researchers have also proposed using backpropagation (BP) in conjunction with STDP. In this way, local learning can be combined with supervision to achieve higher accuracy. For example^27^, the authors apply BP in every time step. The scheme achieves 97.20% accuracy on MNIST using 468,000 synapses in 1200 epochs, each having tens of time steps. A similar example is given in^28^, where convolutional layers generate feature maps, resulting in thousands of spiking neurons in the first layer. The system may also use a second convolutional layer to improve accuracy. The second layer is followed by an SNN having 1500 neurons in the first layer and 10 neurons in the second one. All these neurons are equipped with BP and STDP to achieve high accuracy. The network in^28^ achieves 98.6% accuracy after training for 100s of iterations and multiple time steps. The works in^29^ and^30^ use 100s of feature maps in the first layer followed by a hidden layer having hundreds of neurons. The scheme is unsupervised in nature, and uses a lot of exponential and multiplication elements. The scheme also uses a winner-takes-all (WTA) in the feature discovery layer (last). In^29^, after 20 time steps and 10 training iterations, the scheme successfully achieves around 98.36% accuracy.

To summarize, the poor hardware efficiency inherent in STDP can be attributed to the following: STDP requires way more synapses than BP to achieve a reasonable accuracy; STDP requires more time to train SNNs; STDP requires complex computations. This is why hardware systems based on STDP can neither yield high accuracy, nor are power- or area- efficient.

#### Backpropagation-based direct training schemes

Direct training relies on the use of step function in the forward pass and generally uses surrogate gradient in the backward pass. This is because the exact gradient of the step function is the ‘dirac delta’ function that cannot be used for backpropagation. Typical examples are^4,9,21^.

Though direct training methods that include temporal information for training SNNs (such as STBP^9^, Spike Train-Level BP^31^, and Temporal Spike Sequence Learning (TSSL)^32^) achieve high accuracy, their training cost is high because the method is similar to the one used in recurrent neural networks (RNNs) and cannot extend to complex network infrastructures like VGG-16 and ResNet-20^33^ if high hardware efficiency is required.

A typical example is that of^34^, where the researchers use a 38-layer ResNet. The scheme uses *batch normalization through time* (BNTT), instead of simple batchnorm to improve performance. With 20 time steps and tens of epochs, the system achieves only 92.8% accuracy on CIFAR-10 dataset. The scheme is so complex that it is not appropriate even for hardware-based inference engines, let alone online learning engines.

Similarly, the authors in^35^ admit that direct training schemes are generally not at par with convolutional networks. In order to mitigate the performance degradation, the authors in^35^ propose a direct training scheme that uses an extremely complex gradient function. Since the gradient function uses a large number of multiplications and one division, it is unsuitable for hardware implementations. The accuracy achieved with 8 time steps is 70.2% on CIFAR-10 DVS dataset.

There are several reasons to believe that direct training might not be hardware efficient. One epoch in SNNs is composed of multiple time steps and direct training generally requires an SNN to be unrolled in both space and time, such as in^9^. To the best of our knowledge, the only exception to this rule is demonstrated in^21^, where the time steps are independent but the network is trained using direct training with high accuracy.

Another disadvantage of direct training is that the systems using this method can be used to train SNNs only, unlike ANN-to-SNN conversion methods. This is because the inference part in all these systems allows spikes only. The online learning systems that use ANN-to-SNN conversion, on the other hand, contain computational engines for both the ANN functions and spikes. Moreover, direct training is not suitable for (online) on-chip learning, since the method is extremely complex and requires an enormously large number of multipliers, dividers, storage and exponential elements. The SNN training methods proposed in^9,11^ suffer from similar problems. Therefore, online systems that rely on ANN-to-SNN conversion can be used to train both ANNs and SNNs. Based on all these facts, one may conclude that direct training schemes should be studied carefully before their implementation on hardware.

#### Supervised, temporal learning

Supervised temporal learning schemes generally rely on the use of firing times of output neurons to perform classification. Tempotron is a famous algorithm that *uses the time-to-first-spike* to classify input samples^12^. The algorithm is supervised and hence, more accurate than STDP. However, Tempotron is suitable only for binary classification and cannot be used for multi-class classification. An important hardware system that uses Tempotron for online learning is given in^36^.

In^37^, the authors present a supervised temporal BP method for SNN training. The algorithm uses alpha post-synaptic potential (PSP) function to backpropagate (adjust weights) in such a way that the firing times get adjusted properly. Then, at the output side, the neuron that spikes first corresponds to the classified output. For BP, the algorithm uses Lambert function that is quite complex. In fact, the whole scheme uses a lot of exponentials, dividers, multipliers, etc. that make the algorithm hardware-inefficient and unsuitable for on-chip learning. Moreover, the scheme requires hundreds of time-steps to train. For inference-only systems, the algorithm works fine since it gives an accuracy of around 97.96% for about 269,960 synapses.

The authors in^38^ present a supervised method of SNN training, which they refer to as SpiFoG. The SpiFoG uses evolutionary optimization to train synapses by introducing random synaptic delays. The scheme uses hybrid crossover method for faster convergence.

In^39^, the authors present a supervised, temporal BP method. The researchers highlight a unique problem facing SNN training based on alpha PSP function. According to the authors in^39^, alpha PSP function results in a lot of dead neurons due to its leaky nature. Not only that, since the peak of alpha function is constant, it can cause gradients to explode in the proposed learning scheme. In order to resolve exploding and vanishing gradient issues, the authors proposed a new PSP function ReL-PSP that uses ReLU function for spiking neurons. Since ReLU does not leak, it directly solves the *dead neuron problem* to a great extent. The number of time steps they set for training is around 100. The network they use for performance evaluation (on MNIST) has 317,600 synapses and yields around 98.1% accuracy. The learning scheme does not require complex threshold balancing or regularization of any kind. They train their network off the chip and then perform inference on a popular pre-designed neuromorphic platform YOSO to prove that their scheme is energy efficient. In spite of all these claims, the algorithm is not suitable for on-chip SNN training since it requires extremely complex dividers and other computational elements.

#### ANN-to-SNN conversion schemes

In ANN-to-SNN conversion schemes, a network is first trained in the ANN domain and the trained network is then converted into an SNN. Though the conversion process results in a slight loss, this approach is extremely beneficial because ANN training schemes are very mature and yield high accuracy. Moreover, this approach is suitable for ultra-large networks since it is not as complex as direct training. Important examples from this domain include^13,14,40,41^.

In^14^, the training scheme is based on ANN-to-SNN conversion. The researchers use ReLU at all the hidden layers of a network, and Softmax at the output layer. The rationale behind this approach is that ReLU resembles the input-frequency (IF) curve of a leaky integrate and fire (LIF) neuron. This approach has several drawbacks. First, there is no direct way to convert either ReLU or Softmax into a hard threshold (SNN activation). Secondly, it requires a large time period to get a reasonable accuracy. In order to convert Softmax function in a spiking activation, the firing rate is chosen as the metric; there is no direct/proper way of conversion. Moreover, conversion of ReLU to a spiking neuron requires *weight-threshold balancing*^13,14,40^, which can be quite hectic.

Another example is that of the Nengo platform^42^ that relies on the work presented in^43^. The scheme presented in^43^ uses soft leaky integrate-and-fire (LIF) neurons to enhance differentiability for backpropagation. The researchers add Gaussian noise to the neuronal firing rate to further enhance differentiability. Moreover, post-synaptic filters are added to the neurons to remove high frequency variation produced by spikes. The Gaussian noise is removed from the neurons after training. The basic problem with^43^ is that the algorithm is extremely complex and the accuracy depends on the amount of Gaussian noise and smoothness of LIF neurons. The scheme is, therefore, unsuitable for hardware implementations. Even with a large number of convolutional filters and 30 time steps, the network achieves only 98.74% accuracy on the MNIST dataset.

Therefore, such ANN-to-SNN conversion methods achieve high accuracy but are tricky to apply. An advantage of developing on-chip solutions based on ANN-to-SNN conversion methods is that the resulting system is not only able to train SNNs, but is also able to use both ANNs and SNNs for classification. This is because the inference part of the system contains both artificial and spiking parts.

In^44^, the authors present a BP scheme based on ANN-SNN conversion. The motivation is that traditional ANN-SNN conversion schemes that use ReLU focus only on the conversion of positive voltage region to spikes, since ReLU completely cancels out the negative region. However, this process is lossy because negative regions, sometimes, contain valuable information. The authors solve this problem by using two thresholds for a spiking neuron, one for the positive region and one for the negative region.

- The scheme takes a long time (hundreds of time steps) to train SNNs
- Whether this scheme can yield a reasonable accuracy using a small number of synapses is doubtful. The reason is that the network employed for performance evaluation has about 2.4 million synapses and is able to achieve 98.7% accuracy on MNIST dataset.
- The scheme is not suitable for hardware systems capable of training SNNs since it is extremely complex. The scheme requires thousands of multipliers, and hundreds of dividers and exponent computers.

### SNN-based neuromorphic architectures

Most of the work related to SNNs is for Von Neumann systems only. Only a few researchers have focused on building dedicated hardware systems using SNNs. The most recent example is Akida, developed by BrainChip that contains 1.2 million neurons^45^. Akida is a neural chip that has the capability to process and analyze images, sound, and data patterns. Akida, like many other neuromorphic processors, works in an event-driven manner.

#### Inference-only engines (offline learning)

Most of the SNN processors are built for offline classification. They do not have the ability to train SNNs. Typical examples are^3,19,20,25,46^.

In^25^, the authors train their network using STDP on a computer and then use the obtained parameters on an FPGA. The problem they solve is related to the binary classification of 5$\times$5 samples. The system achieves about 89% accuracy. In^46^, the hardware inference system is based on sigmoidal neurons and predicts whether the incoming sample corresponds to epilepsy or not.

TrueNorth is a chip that was developed in 2014^3^. The computational cores operate at a high frequency but generate their own clock ticks that are entirely dependent on events, and are not awake all the time. This asynchronous behavior is what makes TrueNorth power-efficient. TrueNorth can operate using only 65 mW of power.

Darwin^20^ is another SNN inference system that has only 8 neurons on the chip and keeps reusing those 8 neurons to implement more complex multilayer perceptron (MLP) structures. The system uses STDP for training and uses the address-event representation (AER) protocol that offers a great deal of scalability. Minitaur is a similar example that implements a 784-500-500-10 network on an FPGA^19^. The network is trained on an external computer using STDP. In^41^, the batch normalization (BN) process is integrated into the thresholding process in order to avoid repeated BN calculations. Then, the trained ANN is converted into an SNN.

#### Online learning engines

A big problem associated with most neuromorphic processors is that they can be used only for inference and cannot train SNNs. SNN training is extremely difficult and is not possible to be carried out efficiently on Von Neumann processors (VNPs) due to two main causes: serial execution model followed by VNPs^3^; longer training periods required by SNNs since training depends a lot on the parameter initialization^9^ . Therefore, the development of dedicated processors capable of online (on-chip) learning is an emerging research area.

Various online learning processors have been presented in the literature. Some of the many examples are Adaptive Clock-/Event-Driven Computing System (ACECS)^17,18,36,47–49^. ACECS is a system that is able to switch itself from clock mode to event-driven mode in a dynamic fashion; this feature helps save energy. However, a major problem surrounding all these online learning processors is that they are all based on STDP algorithm, which yields poor classification accuracy. Moreover, STDP is not efficient when it comes to hardware implementations since it requires an enormously large number of synapses and computation of complex terms.

Though the work in^36^ implements Tempotron as the learning algorithm on an FPGA, the work suffers from similar problems: Tempotron requires a large number of exponential terms and other such complex elements, and does not yield high accuracy in case of multiclass classification problems. In fact, Tempotron is designed only for solving simple binary classification problems.

#### Problem definition

Most of the systems based on SNNs support inference only. Only a few SNN systems support online learning. Not only that, since most of the online learning systems use unsupervised learning algorithms such as STDP, they yield poor accuracy and have a high implementation cost. Therefore, the goal is to design an online SNN learning system that is cheap and yields high accuracy.

Keeping all these issues in view, we first present a novel, Hard-Sigmoid-based SNN training (HaSiST) algorithm. HaSiST is a high-performance SNN backpropagation algorithm that is quite efficient for hardware implementations since the activation function requires only shifters and adders. Not only that, it reduces the use of multipliers even in the backward pass to a minimal. We then present a low-cost, high-throughput SNN training engine that uses HaSiST for learning. Since hard sigmoid, just like ReLU, is an ANN activation function, the HaSiST engine can be used for training ANNs as well.

## HaSiST: proposed SNN learning scheme

In this section, we first explain how we perform input/output (I/O) coding, and then present the proposed learning scheme.

### Input coding

In the proposed method, the training is carried out using full resolution inputs to avoid any loss of data. Spikes are generated only in the deployment (SNN) phase. In the deployment phase, the input values are compared against a predefined threshold in every timestep. If the intensity is greater than this predefined threshold, a spike is generated else nothing happens. This method is deterministic in nature since the threshold is fixed for a particular timestep. We avoid the use of Poisson method to generate spikes since it involves a lot of ‘jitter’ and results in a variability in the firing rate^14^. It is pertinent to mention that every timestep corresponds to a different threshold. The threshold remains constant only for one particular timestep. This method results in a much clearer spike map and give much better performance. Not only that, the method is quite hardware friendly since it requires only an array of small comparators and does not depend on random number generation.

### Output coding

At the output side, *one-hot coding* is used, which means that every neuron in the layer belongs to a discrete class. For surrogate network training and inference, we use neuronal membrane potential. For the deployment network (SNN), on the other hand, we use spike rate to perform classification (inference) of input samples. The output neuron that spikes the most corresponds to the predicted class.

### Learning scheme

The proposed learning scheme is based on the conversion of a surrogate network to the deployment network (SNN). In this approach, a surrogate network using a suitable ANN activation function is first trained using backpropagation and the finalized/trained network is then converted into an SNN. The approach, therefore, deals with two networks: the surrogate network and the deployment network.

#### Surrogate network (HaSi)

The purpose of surrogate network is to train a network that is finally going to be converted into an SNN. The neuron model used in the proposed scheme is given in Eq. (1).

1 $$\begin{aligned} V_j[t] = \lambda V_j[t-1] + \sum \limits _{i} \left( W_{i} \cdot X_{i} \right) \end{aligned}$$

All neurons in the surrogate network use hard sigmoid as the activation function. It has the same topology as the final deployment network that is going to be an SNN. Full-resolution inputs are applied to the surrogate network to prevent any loss of information. The generalized form of HaSi function is given in Eq. (2).

2 $$\begin{aligned} A(V_j[t]) = \left\{ \begin{array}{ll} 0 &{} \quad V_j[t] < l_1 \\ \frac{k\left( V_j[t] - V_{th}\right) + 1}{2} &{} \quad l_1 \le V_j[t] \le l_2 \\ 1 &{} \quad V_j[t] > l_2 \\ \end{array} \right. \end{aligned}$$

**Figure 1:**
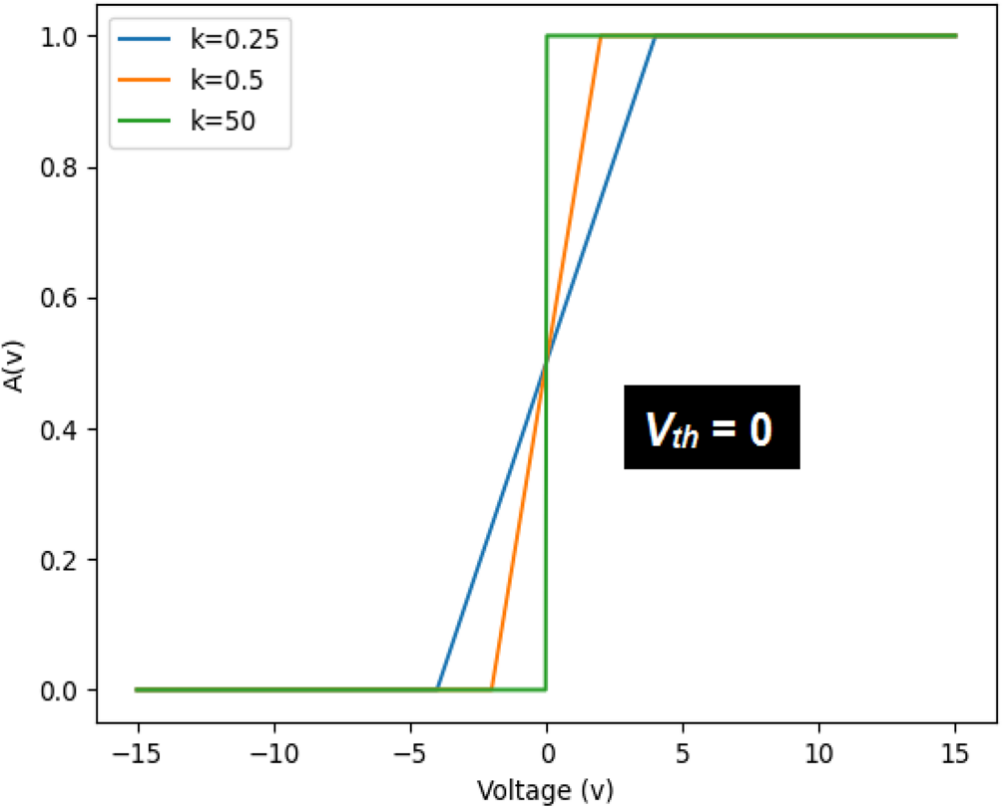
Impact of Steepness Factor *k* on the Shape of Hard Sigmoid.

Here, *k* is the steepness factor that controls the level of steepness of the HaSi; $l_1 (= V_{th} - k^{-1})$ and $l_2 (= V_{th} + k^{-1})$ are the cutoff and saturation points of the HaSi curve. The threshold $V_{th}$ of the hard sigmoid (at least in the proposed work) is its ‘point of inflection’. It is the point at which the activated value is exactly equal to *0.5*. This point is crucial for deciding threshold of a spiking neuron, as mentioned in Section "Deployment Network (SNN)". The impact of steepness factor *k* on HaSi shape for *zero threshold* ($V_{th}=0$) is given in Fig. 1.

#### Deployment network (SNN)

The surrogate network trained using HaSi neurons is finally converted into an SNN. This is done by converting $V_{th}$ of a HaSi neuron to a hard threshold. If the input voltage value falls to the right side of $V_{th}$, the neuron generates a *one*, else it generates a *zero*. This is described mathematically in Eq. (3).

### Analytical and mathematical proofs

HaSiST makes the training process quite simple because the spiking activation function and HaSi are inter-convertible. This is discussed in the following parts.

- *Forward pass* If the steepness factor *k* is equal to $\infty$, the $A(V_j[t])$ in Eq. (2) becomes equal to the step function. This is shown mathematically in Eq. (3).
  3 $$\begin{aligned} A(V_j[t]) = \left\{ \begin{array}{ll} 1 &{} V_j[t] \ge V_{th}^+ \\ 0 &{} V_j[t] \le V_{th}^- \\ \end{array} \right. \end{aligned}$$
- *Backward Pass* In order to perform backpropagation, the activation function must have a finite derivative. Since hard sigmoid is differentiable and has a finite derivative, it can be used for backpropagation. Moreover, unlike ReLU, it can be easily used even at the output layer of a network since it does not cancel out the negative input region completely. The derivative of HaSi is given in Eq. (4).
  4 $$\begin{aligned} A^{'}(V_j[t]) = \left\{ \begin{array}{ll} \frac{\gamma k}{2}; &{} l_1 \le V_j[t] \le l_2 \\ &{} \\ 0 &{} otherwise \\ \end{array} \right. \end{aligned}$$
  Moreover, if *k* is equal to $\infty$, the HaSi derivative becomes equal to the dirac delta function, just like the step function. This is evident from Eq. (5).
  5 $$\begin{aligned} A^{'}(V_j[t]) = \left\{ \begin{array}{ll} \infty &{} V_j[t] = V_{th}^+ \\ 0 &{} V_j[t] != V_{th}^+ \\ \end{array} \right. \end{aligned}$$
  In Eq. (4), the parameter $\gamma$ (gradient controller) is an optional parameter that can be maneuvered to overcome gradient vanishing/explosion. Similarly, $l_1$ and $l_2$ can be changed manually in some cases to overcome gradient vanishing/explosion.
- *Sigmoidal behavior* Both HaSi and the step function are sigmoidal in nature. This is because $A(-\infty )=0$ and $A(\infty )=1$.

### Mathematical setup

We mathematically establish the dependence of loss functions (*L*) on synaptic strengths in Eq. (6) through Eq. (8). The mean squared error (MSE) function has been used for SNN backpropagation. In the following equations, $A_2$ is the obtained output value at Layer 2, and *y* is the label voltage. The derivative of HaSi function is given in Eq. (4) for reference.

6 $$\begin{aligned}{} & {} \frac{\partial L}{\partial W_2} = \left\{ \begin{array}{ll} \frac{\partial L}{\partial A_2[t]} \cdot \frac{\partial A_2[t]}{\partial V_2[t]} \cdot \frac{\partial V_2[t]}{\partial W_2} &{} \quad l_1 \le V_2[t] \le l_2 \\ 0 &{} \quad otherwise \end{array} \right. \end{aligned}$$

7 $$\begin{aligned}{} & {} \frac{\partial L}{\partial W_2} = \left\{ \begin{array}{ll} \frac{k}{2} \cdot \left( A_2[t] - Y[t] \right) \cdot \left[ \lambda \frac{\partial V_2[t-1]}{\partial W_2} + A_1[t] \right] ; \\ \\ \qquad {\textbf {if}} \quad l_1 \le V_2[t] \le l_2 \\ \\ 0 \qquad otherwise \end{array} \right. \end{aligned}$$

8 $$\begin{aligned}{} & {} \begin{aligned} \frac{\partial L}{\partial W_1} = \left\{ \begin{array}{ll} \frac{k^2}{4} \cdot W_2 \cdot \left( A_2[t] - Y[t] \right) \cdot \left[ \lambda \frac{\partial V_1[t-1]}{\partial W_1} + X[t] \right] ; \\ \\ {\textbf {if}} \quad \quad l_1 \le V_1[t] \le l_2 \quad {\textbf {and}} \quad l_1 \le V_2[t] \le l_2 \\ \\ \\ 0 \qquad otherwise \end{array} \right. \end{aligned} \end{aligned}$$

Although all these equations are perfectly valid, temporal dynamics are generally ignored in backpropagation-based SNN training^50^. This is because the notion of ‘time’ does not have a significant impact on the SNN training process. In^9^, for example, the inclusion of time in the SNN training process increases the accuracy by only 0.41%. Moreover, the inclusion of time unnecessarily makes the training process complex and significantly increases hardware cost. To ignore temporal dynamics during the training process (for the surrogate network), we make $\lambda$ equal to *zero*. For the SNN inference, it is up to the user to decide the value of $\lambda$, since it can sometimes improve the performance a little bit. The reason is that the LIF neuron acts as a low-pass filter and blocks high frequency noise in the system^51,52^. The presence of *leak*, therefore, improves system performance in case it encounters high-frequency noise. The dependence of loss function (L) on $W_1$ and $W_2$, therefore, is given in Eqs. (9) and (10) respectively.

9 $$\begin{aligned}{} & {} \frac{\varvec{\partial } \varvec{L}}{\varvec{\partial }\varvec{W}_{\varvec{1}}} = \left\{ \begin{array}{ll} \frac{ k^2 \overrightarrow{W_2} \left( \overrightarrow{A_2} - \overrightarrow{Y} \right) \overrightarrow{X} }{4}; \\ \\ {\textbf {if}} \quad l_1 \le V_1 \le l_2 \quad {\textbf {and}} \quad l_1 \le V_2 \le l_2 \\ \\ \\ 0 \qquad otherwise \end{array} \right. \end{aligned}$$

10 $$\begin{aligned}{} & {} \frac{\varvec{\partial }\varvec{L}}{\varvec{\partial }\varvec{W}_{\varvec{2}}} = \left\{ \begin{array}{ll} \frac{ k \left( \overrightarrow{A_2} - \overrightarrow{Y} \right) \overrightarrow{A_1} }{2}; &{} l_1 \le V_2 \le l_2 \\ \\ 0 &{} otherwise \end{array} \right. \end{aligned}$$

The system performance can slightly be improved if network regularization, dropout, and optimization (such as Adam^53^) are performed. However, all such methods will result in an unnecessary (disproportionate) increase in cost and power consumption since they are costly to implement on hardware. Moreover, they do not offer any significant increase in network accuracy. Therefore, we do not adopt any such technique/method because the whole learning process is performed on hardware.

## Proposed neuromorphic computer

The proposed hardware system operates on 64-input data samples and can classify an input sample into one of ten classes. The system consists of a timer, a counter, a scheduler, an inference engine, and a training engine. The training engine is executed only when network training is required, else a sample is classified using pre-stored weights and incoming external inputs.

**Figure 2:**
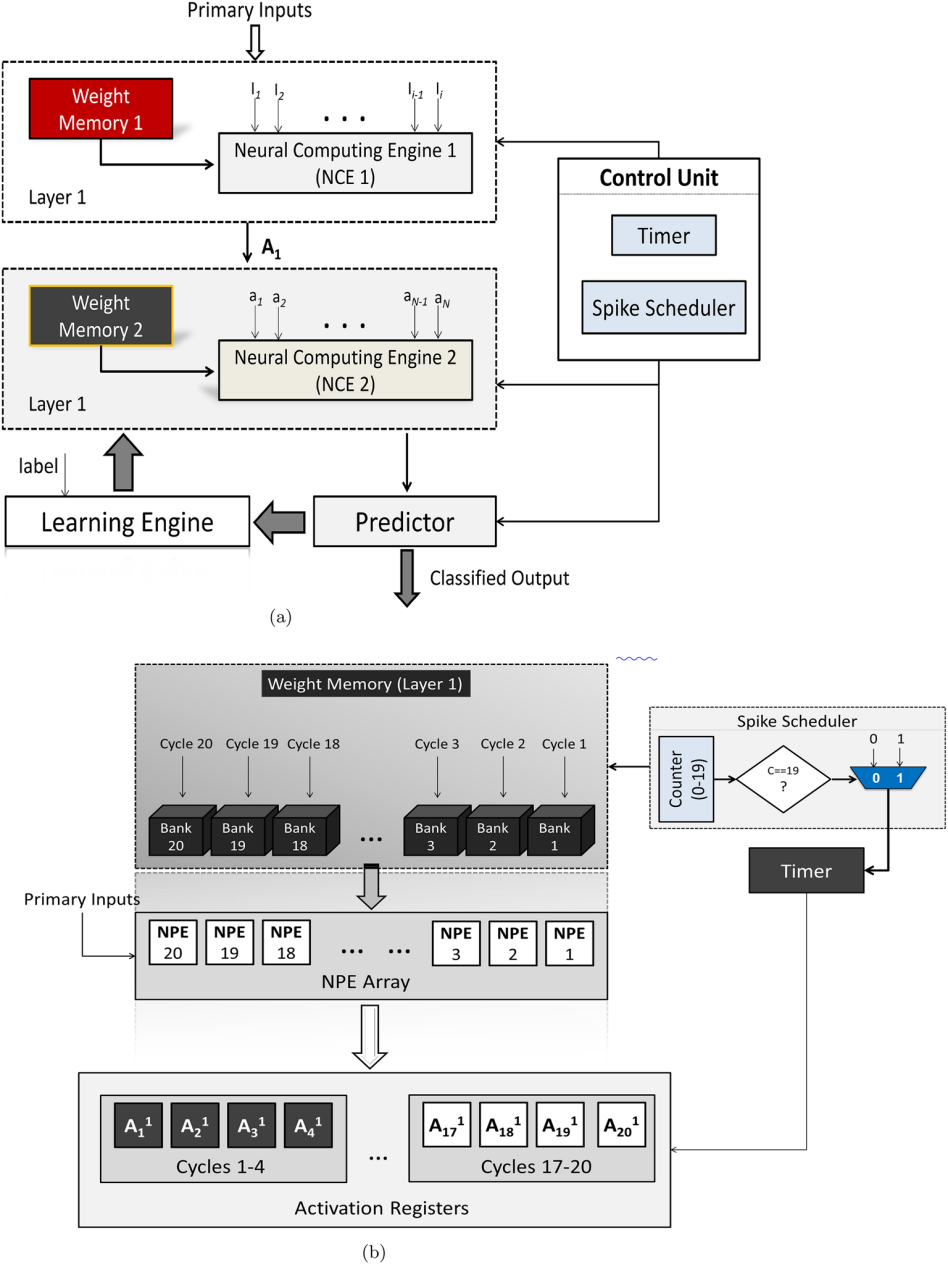
Internal Structure of the Proposed System. (**a**) Top Level Diagram of the Proposed System. (**b**) Internal Structure of the Layer 1 Neural Computing Engine.

The proposed hardware-based neural network has one input layer, one hidden layer and one output layer. The hidden layer has 20 neurons that are processed sequentially. Only one hidden neuron is activated in a given clock cycle in order to reduce hardware cost. The top level diagram of the complete system is shown in Fig. 2a.

### Proposed spike scheduler and router

Most scalable SNN processors use the following elements to transmit and route packets from a source neuron to a destination neuron: source neuron address, target neuron address, and timestamp^17^. However, there are some serious flaws in this approach. First, it is not necessarily suitable for low-cost systems because all the neuron addresses require a large number of bits and complex computational elements, as a result of which the cost of transmission and routing becomes very high. Moreover, it is not suitable for surrogate-network used for SNN training. This is because the (surrogate) training network is never driven by events since training is carried out using continuous activation function such as ReLU (hard sigmoid, in the proposed scheme). Therefore, neuron addresses are not required to process anything.

The alternative approach, as adopted in the proposed system, is *universal broadcasting*. In this scheme, all the source actuators transmit their output values to all the connected destination neurons. This approach simply obviates the need for large spike/activation packets that require source neuron address, destination neuron address, and timestamp. This approach is similar to the one presented in^17^ and works well for moderately large networks.

### Timer and counter

The timer is realized using a $\log_2(T)$ digital counter. The timer is incremented by 1 once all the neurons have completed their respective processes, i.e., the timer is incremented at the end of 21 computational clock cycles. This is because Layer 1 neurons complete their computations in 20 cycles and one cycle is consumed by Layer 2. The timer keeps counting up until it reaches the maximum time period ‘T’, after which it is reset.

### Reconfigurable inference engine (RIE)

The inference engine is responsible for classifying the input sample into one of the ten classes. It does so by using two things: weights (stored in the on-chip memory), and inputs (coming from external memory).

During the forward pass, the network can operate in one of two modes: HaSi Mode or SNN mode. In the HaSi mode, hard sigmoid is used for inference, whereas hard threshold (SNN activation function) is used in the SNN mode. It is pertinent to mention that training can be performed only in the HaSi mode.

#### Multiplier–accumulator (MAC) unit

For HaSi inference, full-resolution inputs are multiplied by the corresponding weights. In the SNN mode, 1-bit spikes are used as selection bits to the multiplexer. If the selection/spike bit $s_n$ is 0 which implies that no event has taken place, no weights are fetched from the memory and a 0 is passed to the subsequent stages. If the spike bit is 1, which implies an event, the corresponding weight is fetched from the memory and is passed to the subsequent stages. This is the job of neural processing elements (NPEs), shown in Fig. 2b .

The obtained values are then summed up using a pipelined adder tree and the final value is then passed through either the hard sigmoid function or the SNN activation function, depending on the mode selected by the user. The activated values are then sent to the second-layer RIE to perform similar operations in Layer 2. Layer 2, unlike Layer 1, is fully parallelized and all operations in Layer 2 are performed in a single clock cycle.

**Figure 3:**
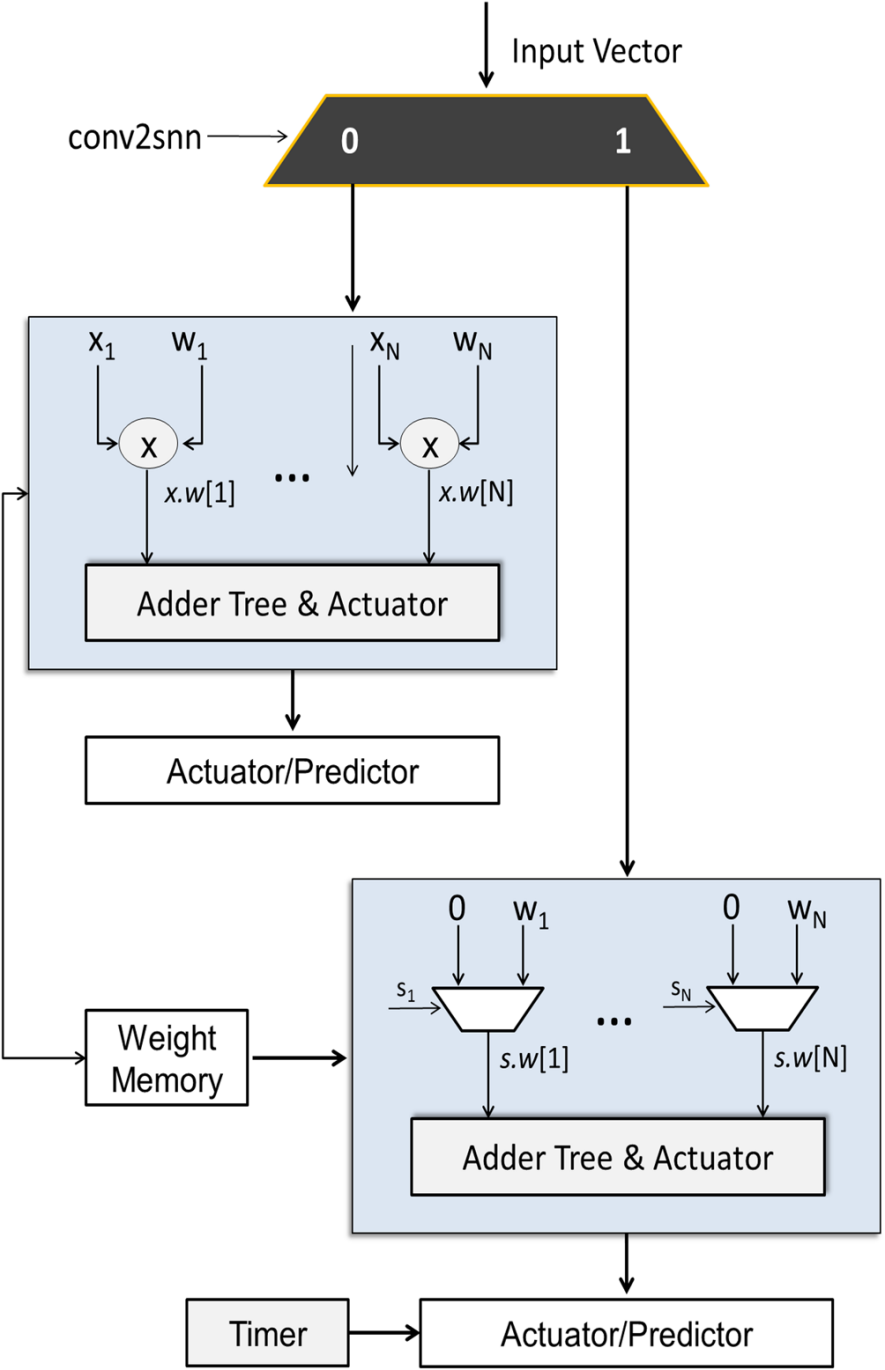
Reconfigurable Inference Engine.

#### Actuators

Actuators are responsible for activating a neuron. There are two types of actuators available in the proposed engine: HaSi actuators and SNN actuators.

SNN actuator operation is quite simple to understand: if the incoming voltage is greater than a threshold, a spike is generated, else there is no spike. In fact, if the threshold is *zero*, only the most significant bit of the incoming voltage bus has to be compared in order to determine whether the voltage is greater than *zero*. The reconfigurable engine is shown in Fig. 3.

HaSi actuators, on the other hand, are slightly more complex. The actuator is constructed using a priority routing network, which is quite effective in implementing complex control instructions. Here, if the voltage is greater than 2, the actuator saturates the value to 1. If the voltage is too small, i.e., less than 2, the actuator simply transfers a *zero* to the subsequent circuitry. If the incoming value lies between these two extremes, the incoming voltage is first added to a value (2 in our case) and then divided by a value (4 in this case). In the proposed system, this division by 4 is realized using a 2-bit right shifter.

#### Predictor

The predictor is the last component of the system that sits at the output layer. The sole purpose of predictor is to compare the output (activated) values with each other and make a decision.

In the HaSi mode, temporal information can simply be rejected, as mentioned earlier. Therefore, there is no role of timer as such in the HaSi mode. In the HaSi mode, the output neuron with the maximum level of voltage is chosen as the predicted/classified output.

In the SNN mode, however, temporal information is of utmost importance to make a decision. Therefore, the timer does play its role in the SNN mode. Once the input time period is over, the predictor makes a decision on the basis of number of spikes. In the SNN mode, the output neuron with the maximum number of spikes is chosen as the predicted output. This is what it means by ‘rate coding’, because the rate of firing/spiking is being used to make predictions.

### Training/learning engine

**Figure 4:**
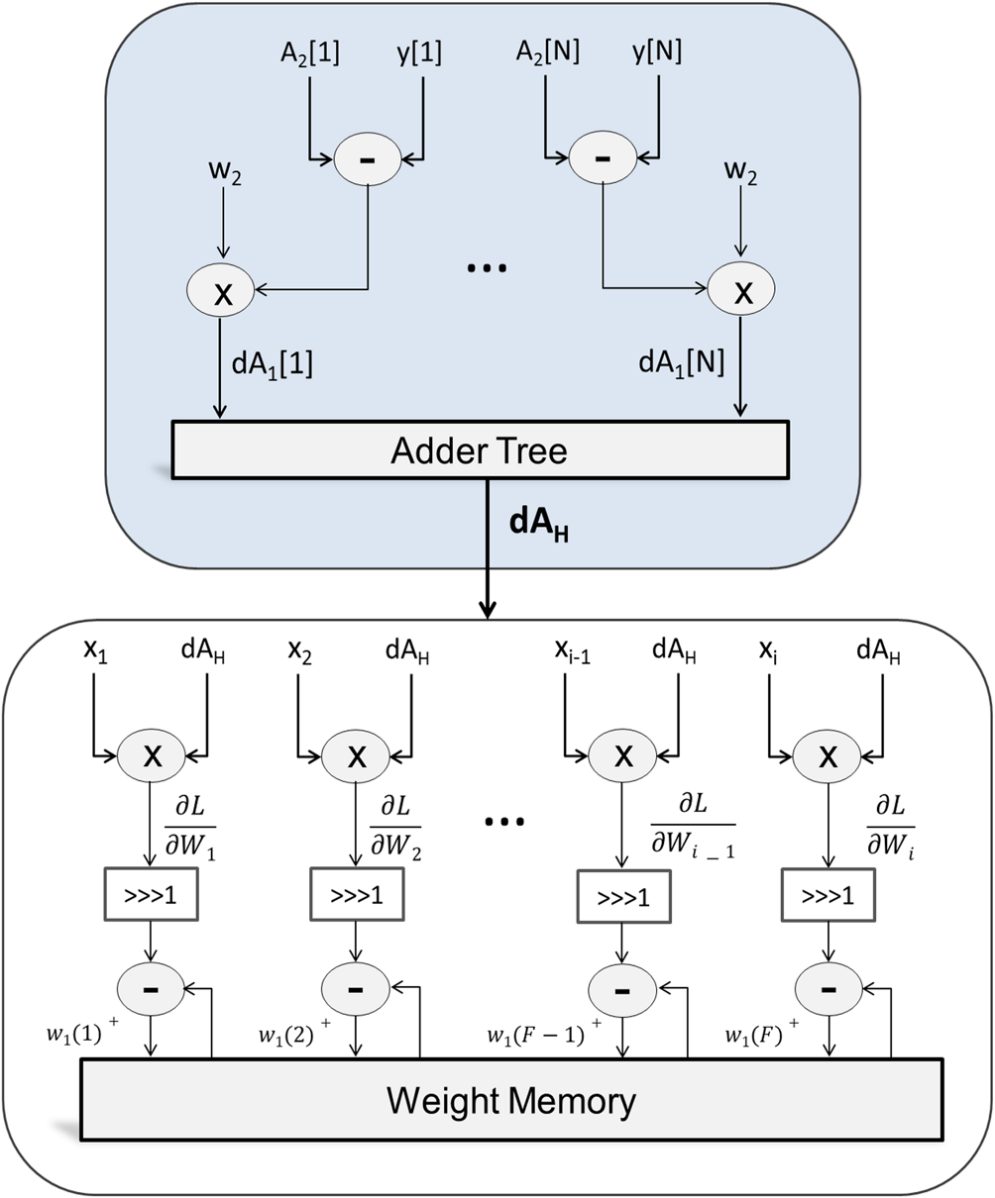
Internal Structure of the Layer 1 Learning Engine (Backward Pass).

The learning engine employs backpropagation on surrogate network to learn network parameters. The learning takes place according to Eq. (9) and Eq. (10). The structure of the learning engine used for training Layer 1 (L1) is shown in Fig. 4. The letters *F* and *X* in Fig. 4 denote the feature vector and input vector, respectively. The learning rate is realized using a 1-bit right shifter since it is equal to 1/2. It can be clearly seen in the figure that the learning can take place without any costly exponentials and/or dividers. Using only a few multipliers and adders, the engine can train the network.

#### Train-while-constrain (TWC) approach

The approach we follow is *train-while-constrain*, which simply means that we train the network while we constrain the intermediate computational results to an acceptable level during the training process. The purpose of overflow/underflow detector is to keep the number of bits to a controllable level so that the computational units operate properly, and the hardware cost does not cross a certain limit. In the proposed hardware engine, 8-bit weights and 7-bit actuators are used.

## Results

In this section, we first detail the test conditions under which the performance evaluation was carried out and then present the obtained results. To compare systems in a fair manner, the proposed system is compared against various modern systems in terms of both cost and throughput.

### Benchmarks and test conditions

The software tool used for performance evaluation is Python. Two popular datasets have been used for algorithmic evaluations: *MNIST*^54^ and *8*$\times$*8 Digits*^55^. The test conditions and hyperparameter values used for all such evaluations are mentioned in Table 1. The hyper-parameter values have been empirically obtained using ‘grid search’.

**Table 1:** Hyper-parameter values obtained from network tuning.

Parameter	Value	
	8$\times$8 Digits	MNIST
Learning Rate ($\eta$)	$\frac{1}{8}$ - $\frac{1}{2}$	1
Topology	64-20-10	784-200-10
Batch Size ($\xi$)	1	100
Momentum ($\beta$)	0	0.9
Epochs	64–400	18
$(l_1, l_2)$ (Hid. Layer)	$-2$, 2	$-2$, 2
$(l_1, l_2)$ (Out. Layer)	$\le -2$, $\ge$2	$-20$, 20
$\gamma _{hid.}$	1–2	1
$\gamma _{out.}$	1–2	1–2
Surr. Leak Factor ($\lambda$)	0	0
Depl. Leak Factor ($\lambda$)	2$^{-6}$	0
Input Encoding	Thresholding	Thresholding
#TimeSteps (*T*)	15	10
Input Thresholds	0–14	10, 30, ..., 160, 190, ..., 240
Neural Encoding	Rate-based	Rate-based
HaSi Thresh. ($V_{th}$)	0	0
Steepness (*k*)	0.5	0.5

The hardware system is described and verified using Verilog language at the register-transfer level (RTL) for the target system Virtex 6. To show the hardware efficiency of the proposed system, the *8*$\times$*8 Digits* has been used. This dataset has 64 input features and 10 outputs. The system contains 20 hidden neurons.

### Network accuracy comparisons

The accuracy yielded by HaSiST on the two datasets, namely MNIST and 8$\times$8, is 97.5% and 99% respectively, ignoring the impact of truncation. A comparison of HaSiST with various modern schemes in terms of inference time, accuracy and the required number of synapses is given in Table 2.

**Table 2:** Accuracy Comparisons - MNIST.

	Acc. (%)	Algorithm	Synapses	#TimeSteps
^13^	98.68%	STDP	2,392,800	6–10
^21^	98.70	STE-BP	1,861,632	16
^9^	98.89	STBP	635,200	30
^41^	98	STBNN	1,794,000 (1-b)	50
^56^	90	Sup. STDP	13,300	–
^37^	97.96%	TBP	269,960	100 s
^39^	98.1	TBP	317,600	100
^44^	98.7	BP	>2.4 Million	100 s
Proposed	$\approx$97.5	HaSiST	158,800	10

### Evaluation of the proposed SNN inference engine

In order to properly evaluate performance and efficiency of the proposed learning scheme and hardware architecture, the inference engine has been synthesized separately as well. The results are tabulated in Table 3. In order to do this, the network is first trained using HaSiST on a personal computer. The obtained weights are converted into 10-bit fixed-point format and are then stored in the FPGA memory. The structure and timing diagram of the inference part has already been described in Section "Proposed Neuromorphic Computer".

The computational frequency of the proposed spiking engine is around 135.073 MHz. A sample of the dataset under consideration contains 64 features. It takes 21 cycles to infer a timestep, and 15 timesteps constitute an SNN sample in the forward pass. It can be concluded, therefore, that the proposed SNN inference engine can infer about 0.03$\times$10$^9$ features in a second. The metric ‘features per second (FeaPS)’ is used for throughput (TP) comparisons since different datasets have different input features. Some datasets have less than 10 features, while others have more than 700. The use of FeaPS results in a relatively fair comparison.

#### Details of the systems used for comparison

It is to be noted that due to the differences in platforms, datasets and other characteristics, an absolutely fair comparison is impossible to be made between various hardware systems.

The work in^25^ uses a small (toy) dataset with 25 binary input pixels and one neuron for binary (X and O) classification; two samples are used for training. The work in^46^ predicts epilepsy; it uses a small number of features and 3 output classes. No dataset is used in^57^; the authors just demonstrate the efficiency of hardware radial basis function. The works in^58,59^ use MNIST for testing purposes.

**Table 3:** Cost and throughput comparisons: inference engines.

System	Acc.	Algorithm	Max. throughput (Features per sec.)	Neuron	Regs./Synapse	LuTs/Synapse	Max. Freq.	Platform
^25^	89%	STDP	4.73$\times 10^9$	e-LIF	7.87	87.22	189 MHz	Virtex 6
^58^	98.32%	Backpr.	0.0023$\times 10^9$	e-LIF	0.014	0.005	250 MHz	Arria-10
^59^	97.06%	Backpr.	0.00013$\times 10^9$	e-LIF	0.004	0.0029	200 MHz	Zynq ZC706
^46^	95.14%	Backpr.	0.25$\times 10^9$	Sigmoid	1.1875	135.11	50 MHz	Altera D2-115
^57^	–	–	39.376	Radial	197.25	298.25	9.844 MHz	Spartan 3
Average	94.88%	–	~1 × 10^9^	–	41.26	104.12	139.77 MHz	**-**
Proposed	>98%	HaSiST	0.03$\times 10^9$	LIF	1.03	2.8	135.1 MHz	Virtex 6

### Evaluation of the proposed SNN learning engine

Table 4 compares HaSiST learning engine with other contemporary engines implementing online learning. The complete learning engine includes both the forward and the backward pass. The learning engine has a maximum frequency of 50 MHz and it takes 21 cycles to complete a training iteration, the engine can perform 2.38 million training iterations in a second.

It can be seen from Table 4 that HaSiST clearly surpasses other contemporary engines in terms of both throughput and cost. At the same time, it can yield a high level of classification accuracy. This can be attributed to the proposed backpropagation-based learning mechanism that yields high accuracy and requires fewer synapses than timing-based and/or unsupervised algorithms. The systems in^23,47,48,60^ are all trained using STDP that does not yield high accuracy and requires millions of synapses. The backpropagation-based learning, on the other hand, uses only a fraction of synapses and can yield excellent accuracy.

#### Details of the Systems used for Comparison

The work in^47^ uses a small (toy) dataset with 25 binary input pixels and 10 neurons for digits classification; the number of training samples is around 10. The work in^36^ uses a small dataset with 4 input features; the purpose is to classify an input pixel into Red, Green, or Blue. The work in^46^ predicts epilepsy; it uses a small number of features and 3 output classes. No dataset is used in^48^ and^61^. The system in^48^ uses STDP for bimodal distribution. The purpose of^61^ is just to demonstrate the efficiency of the hardware learning engine. The work in^49^ uses toy datasets with less than 10 input features and around 3 output classes. The works in^60^ uses MNIST for testing purposes. The system in^23^ uses a context-dependent task for testing; the number of input features are quite small. Two datasets are used for evaluation in^62^: UWB antenna set whose topology is 6-30-2, and an 8-15-8 dataset. The system uses Quasi Newton Optimzation.

**Table 4:** Cost comparisons: online learning engines.

System	Acc.	Algorithm	Neuron	Regs./Synapse	LuTs/Synapse	Max. Freq.	Platform
^61^	–	–	Sigmoid	–	1569	50 MHz	Cyclone IV
^60^	87.7%	STDP	LIF	0.08	0.114	120 MHz	Virtex 6
^62^	$\approx$98%	Quasi-Newton	–	1777	1240	250 MHz	N-FPGA SUME
^23^	90%	STDP	LIF	41.7	284	143 MHz	Kintex-7 XC7kt160t
^36^	96%	Tempotron	LIF	38.1	96.26	178 MHz	Virtex 7
^48^	-	STDP	Izhik.	354.4	518.8	84 MHz	Spartan 3
^49^	88.3%	Backprop.	Sigmoid	80.55	155.5	Variable	Virtex 5
^47^	94%	STDP	Izhik.	33	19	N.A.	Cyclone V
Average	92.3%	**-**	–	332.12	485.33	137.5 MHz	**–**
HaSiST (org.)	99% (98%)	Backprop.	HaSi (Spiking)	-	–	–	-
HaSiST (TWC)	$\approx$98% (95.622%)	Backprop.	HaSi (Spiking)	2.63	37.84	50 MHz	Virtex 6

## Conclusion

This work presents a hardware-aware SNN backpropagation scheme HaSiST that does not require weight-threshold balancing, error normalization, etc. The learning scheme offers fast convergence and easily achieves an accuracy of around 97.5% on MNIST dataset using only 158,800 synapses.

The training engine can operate at a maximum clock frequency of 50 MHz and consumes only 2.63 slice registers and 37.84 look-up tables per synapse. Moreover, the inference engine trained using HaSiST, if run independently, requires only 1.03 slice registers and 2.8 look-up tables per synapse. The inference engine can process about 0.03$\times 10^9$ features per second (FPS), equivalent to about 9.44 giga synaptic operations per second (GSOPS). Here, one synaptic operation is equal to one multiply-accumulate (MAC) operation. If the system is made to operate at an algorithmic clock frequency of 1 kHz, the spiking frequency of a single neuron is around 23 Hz. The design is way cheaper than most online learning engines based on STDP.

HaSiST can yield higher throughput if implemented on a bigger, more powerful FPGA. Moreover, the accuracy might be improved if dropout and batch normalization are used.

## Data Availability

There are two datasets used for experimentation in this work: MNIST and 8x8-Digits. Both are accessible through the Python libraries, and are available publicly from the following sources. MNIST Source: https://www.tensorflow.org/api_docs/python/tf/keras/datasets/mnist/load_data 8x8 Source: https://scikit-learn.org/stable/modules/generated/sklearn.datasets.load_digits.html All the parameters/hyper-parameters used for data processing and experimentation have been mentioned clearly in the manuscript. Table 1 contains all the hyper-parameter values required to replicate the results presented in this work.
